# Effect of *Alpinia katsumadai* Hayata on House Dust Mite-Induced Atopic Dermatitis in NC/Nga Mice

**DOI:** 10.1155/2012/705167

**Published:** 2012-10-02

**Authors:** Hye-Sun Lim, Chang-Seob Seo, Hyekyung Ha, Hoyoung Lee, Jun Kyung Lee, Mee-Young Lee, HyeunKyoo Shin

**Affiliations:** ^1^Basic Herbal Medicine Research Group, Korea Institute of Oriental Medicine, Daejeon 305-811, Republic of Korea; ^2^Constitutional Medicine & Diagnosis Research Group, Korea Institute of Oriental Medicine, Daejeon 305-811, Republic of Korea; ^3^Department of Medical Supplies Development, Hanpoong Pharm & Food Co. Ltd., Jeonju 561-841, Republic of Korea

## Abstract

We evaluated the effects of *Alpinia katsumadai* Hayata (AKH, Zingiberaceae) extract on the production of nitric oxide (NO) and prostaglandin E_2_ (PGE_2_) in RAW 264.7 cells, thymus- and-activation-regulated chemokine (TARC/CCL17) in HaCaT cells, and histamine level in HMC-1 cells. In an *in vivo* experiment, atopic dermatitis was induced by topical application of house dust mites for 4 weeks, and the protective effects of AKH was investigated by measuring the severity of the skin reaction on the back and ears, and plasma levels of immunoglobulin E (IgE) and histamine. AKH extract suppressed the production of NO and PGE_2_ in RAW 264.7 cells, TARC in HaCaT cells, and histamine in HMC-1 cells in a dose-dependent manner. In *in vivo* experiments, the severity of dermatitis, including erythema/hemorrhage, edema, erosion and scaling, and plasma levels of IgE, and histamine were lower in NC/Nga mice with atopic dermatitis, treated with AKH extract than in untreated mice. AKH extract reduced the histological manifestations of atopic dermatitis-like skin lesions such as erosion, hyperplasia of the epidermis and dermis, and inflammatory cell infiltration on the skin of the back and ear. These results suggest that AKH inhibits the development of house dust mite-induced atopic dermatitis in NC/Nga mice.

## 1. Introduction

Atopic dermatitis (AD) is a chronically relapsing, pruritic, and eczematous skin disorder accompanying allergic inflammation. AD is one of the most common diseases, affecting 10–20% of people worldwide [1, 2]. AD can be caused by the interaction of genetic, pharmacological, and environmental factors, resulting in an imbalance within the immune system [3]. Recent immunological analyses of the pathogenesis of AD have produced complex information showing that AD is best characterized as an inflammatory reaction in the skin and a disrupted skin barrier. Until recently, these two factors have been studied as separate entities. However, inflammatory cytokines are known to regulate filaggrin, an important component of the skin barrier, as well as proteins involved in the processing and maturation of filaggrin. Therefore, inflammation itself may induce a functional skin barrier dysfunction and thereby aggravate the eczematous reaction in AD [4–6]. Previous studies have shown that AD is associated with increased serum immunoglobulin E (IgE) levels, increased Th2 cytokine level in the lesional skin, increased serum eosinophil count, and increased expression of proinflammatory enzymes such as iNOS and COX-2. AD also occurs frequently in response to environmental allergens such as the house dust mite *Dermatophagoides farinae (D. farinae) *and 1-chloro-2,4-dinitrobenzene [7–9].

NC/Nga mice have been studied most extensively as an animal model of AD. These mice develop AD-like eczematous skin lesions spontaneously when kept in conventional housing with uncontrolled air but not when maintained under specific pathogen-free conditions. Given the similarity between the clinical symptoms displayed by NC/Nga mice and AD in humans, models based on these mice are thought to provide important information about AD. Repeated exposure to *D. farinae* evokes AD-like skin lesions in NC/Nga mice under specific pathogen-free conditions [10, 11]. 

*Alpinia katsumadai* Hayata (AKH) is used traditionally as an herbal medicine in China and Korea. Previous studies demonstrated that AKH contains a variety of active compounds such as cardamonin, pinocembrin, and alpinetin. Both *in vivo* and *in vitro* experiments have shown that AKH exhibits various pharmacological effects including anti-inflammatory, antioxidant, antimicrobial, and antiasthmatic activities [12–15]. However, no previous work has investigated the effect of AKH on AD or the underlying mechanism.

We investigated the effects of AKH on nitric oxide (NO) and prostaglandin E_2_ (PGE_2_) production in RAW 264.7 macrophages and thymus- and-activation-regulated chemokine (TARC/CCL17) production in HaCaT keratinocytes. We examined the effects of AKH extract on NC/Nga mice as a model of *D. farinae*-induced AD. We measured the skin severity score, histological changes in skin including mast cell infiltration, and plasma IgE, and histamine levels.

## 2. Materials and Methods

### 2.1. Reagents and Materials

Alpinetin and cardamonin were purchased from ChromaDex (Santa Ana, CA, USA). Pinocembrin was obtained from Fluka (St. Louis, MO, USA). The purity of the three compounds was determined to be >98% by HPLC analysis. HPLC-grade reagents, methanol, acetonitrile, and water were obtained from J. T. Baker (Phillipsburg, NJ, USA). 

The seeds of AKH were purchased from HMAX (Jecheon, Korea) in October 2008. These materials were confirmed taxonomically by Professor Je-Hyun Lee of Dongguk University, Korea. A voucher specimen (AK-2008-ST26) has been deposited at the Basic Herbal Medicine Research Group, Korea Institute of Oriental Medicine. The sample was ground to a fine powder and passed using a standard sieve (No. 30, 600 *μ*m) before analysis.

Biostir-AD, an ointment containing extract of the house dust mite,* D. farinae*, was purchased from Biostir, Inc. (Kobe, Japan). A 0.1% ointment of tacrolimus (Protopic; Astellas, Grand Island, NY, USA) was used as a positive control.

### 2.2. Chromatographic System

We used a Shimadzu LC-20A HPLC system (Shimadzu Co., Kyoto, Japan) comprising a solvent delivery unit, an online degasser, an auto sampler, and a PDA detector. The data processor used was LC Solution software (version 1.24, Shimadzu Co., Kyoto, Japan). The analytical column used was a Gemini C_18_ (250 × 4.6 mm; particle size 5 *μ*m, Phenomenex; Torrance, CA, USA). The mobile phases were solvent A (H_2_O) and solvent B (acetonitrile). The gradient flow was as follows: 0–10 min, 30–40% B; 10–30 min, 40–70% B; 30–35 min, 70% B; 35–40 min, 70–30% B, 40–50 min, 30% B. The temperature of the column oven was maintained at 40°C. The analysis was performed at a flow-rate of 1.0 mL/min with PDA detection at 280 nm and 340 nm. The injection volume was 10 *μ*L.

### 2.3. Preparation of Standard Solution

**Figure 1:**
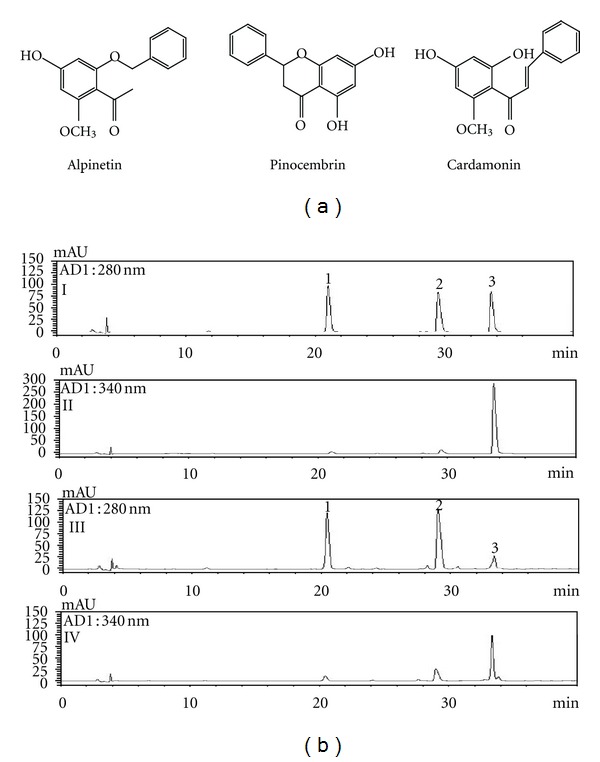
Chemical structures (a) and HPLC chromatogram (b) of reference standards with detection at 280 nm (I) and 340 nm (II), AKH extract at 280 nm (III) and 340 nm (IV).Alpinetin (**1**), pinocembrin (**2**), and cardamonin (**3**).

Standard stock solutions of three flavonoids, alpinetin, cardamonin, and pinocembrin (Figure 1(a), all at 500 *μ*g/mL) were prepared in methanol and kept below 4°C. Working standard solutions were prepared by serial dilution of stock solutions with methanol. All calibration curves were obtained by assessment of the peak areas from standard solutions in the concentration range of 6.25–200.00 *μ*g/mL for alpinetin and pinocembrin, and 3.13–100.00 *μ*g/mL for cardamonin. Each calibration curve was obtained three times, each using eight different concentrations.

### 2.4. Preparation of Sample Solution

The dried seeds of AKH (1.0 g) were dissolved in 100 mL of 70% methanol, and the mixtrre was sonicated for 60 min at room temperature. The solution was filtered through a SmartPor GHP syringe filter (0.2 *μ*m pore size; Woongki Science, Seoul, Korea). The injection volume for HPLC analysis was 10 *μ*L.

### 2.5. Cell Culture

The murine macrophage RAW 264.7 cell line was obtained from the American Type Culture Collection (ATCC, Rockville, MD, USA). The human keratinocyte cell line HaCaT and human mast cell line HMC-1 were kindly provided by Prof. Na Kyung Lee (Sejong University, Seoul, Korea) and Prof. Hyun-Soo Bae (Kyunghee University, Seoul, Korea), respectively. The cells were cultured in Dulbecco's modified Eagle's medium (Gibco Inc., Grand Island, NY, USA) supplemented with 5.5% (Raw 264.7) or 10% (HaCaT) heat-inactivated fetal bovine serum (Gibco Inc., Grand Island, NY, USA), penicillin (100 U/mL), and streptomycin (100 *μ*g/mL) in a 5% CO_2_ incubator at 37°C. The HMC-1 cells were cultured in Iscove's modified Dulbecco's medium (Gibco) supplemented with 10% heat-inactivated fetal bovine serum (Gibco), penicillin (100 U/mL), streptomycin (100 *μ*g/mL) in a 5% CO_2_ incubator at 37°C.

### 2.6. Measurement of NO and PGE_2_ Production

RAW 264.7 cells were plated at a density of 2.5 × 10^5^ cells/well in 48 well plates and incubated overnight. Cells were treated with LPS (1 *μ*g/mL) in the presence or absence of various concentrations of AKH extract. After incubation for 18 h, supernatants were analyzed for the levels of NO (Griess Reagent System; Promega Corp., Madison, WI, USA) and PGE_2_ (ELISA; Cayman Chemical Co., Ann Arbor, MI, USA) according to the manufacturers' protocols.

### 2.7. Measurement of TARC and Histamine Production

To measure TARC and histamine production, HaCaT cells (1 × 10^6^ cell/well) and HMC-1 cells (2 × 10^5^ cell/well) were cultured in 6 well plates and 48 well plates, respectively, with medium containing 10% fetal bovine serum. After reaching confluence, HaCaT cells were washed and then treated with AKH extract in 1 mL of serum-free medium containing TI (each 10 ng/mL; R&D Systems Inc., Minneapolis, MN, USA) for 24 h and HMC-1 cells were treated with AKH extract in 0.5 mL of medium containing Phorbol 12-myristate 13-acetate (PMA, Sigma-Aldrich, St Louis, MO, USA) and calcium ionophore A23187 (Sigma-Aldrich) for 24 h. The supernatants were harvested, and TARC and histamine production were quantified using an ELISA according to the protocol provided by R&D Systems and Oxford Biomedical Research (Oxford, MI, USA), respectively. 

### 2.8. Experimental Animals

Male NC/Nga mice (8 weeks old) were purchased from Central Laboratory Animal Inc. (Seoul, Korea). The animals were housed in an air-conditioned room and maintained at 24 ± 2°C and 55 ± 15% humidity. All experimental procedures involving animals were conducted in accordance with the NIH Guidelines for the Care and Use of Laboratory Animals and approved by Korea Institute of Oriental Medicine Institutional Animal Care and Use Committee. The animals were cared for in accordance with the dictates of the National Animal Welfare Law of Korea.

### 2.9. Induction of Allergic Dermatitis in NC/Nga Mice

AD-like skin lesions were induced in 10-week-old male NC/Nga mice using Biostir-AD, as described previously [8]. Briefly, the hair on the upper back was shaved, and 200 *μ*L of 4% (w/v) sodium dodecyl sulfate was applied to the shaved dorsal skin and both surfaces of each ear for barrier disruption. After 2 h, 50 mg of Biostir-AD was applied topically twice weekly for 4 weeks. The AKH extract and Protopic were each suspended in distilled water and administrated orally to mice. At the start of the experiment, mice were randomized to one of four groups of five mice each: untreated controls (distilled water; control, p.o.), Biostir-AD treated (50 mg/mouse), Protopic treated (50 mg/mouse, topical application), and AKH extract-treated (10 mg/mouse, p.o.). The mice were sacrificed under anesthesia with pentobarbital (200 *μ*g/mouse, i.p.) on the day of the experiment. At autopsy, blood was collected from the posterior *vena cava*, and the back skin and each ear were excised for histopathological analysis.

### 2.10. Evaluation of Dermatitis Severity

The relative dermatitis severity was assessed macroscopically using the following scoring procedure. The severity of dermatitis was assessed macroscopically according to the Eczema Area and Severity Index scoring system: 0, no symptoms; 1, mild symptoms; 2, moderate symptoms; 3, severe symptoms. The dermatitis score was defined as the sum of scores for erythema/hemorrhage, edema, excoriation/erosion, and scaling/dryness [9]. The mice were photographed once per week.

### 2.11. Histopathology

After sacrifice, the back skin and one ear of each mouse were fixed in 10% (v/v) natural buffered formalin for 24 h at 4°C. Tissue samples were embedded in paraffin and thin-sectioned (4 *μ*m thickness), and the sections were stained with hematoxylin and eosin (H&E) solution (Sigma-Aldrich, St. Louis, MO, USA) and mounted under coverslips using Dako-mounting medium (DakoCytomation, Glostrup, Denmark). The stained sections were photographed using a Photometrics Quantix digital camera, and montages were assembled using Adobe Photoshop. To measure mast cell infiltration, skin sections were stained with toluidine blue, and the numbers of mast cells in four chosen sites were counted.

### 2.12. Measurement of Plasma IgE and Histamine Level

Blood samples were drawn from mice, and the plasma was separated by centrifugation at 10000  ×g for 10 min at 4°C and stored at –80°C. The plasma levels of histamine (Oxford Biomedical Research, Oxford, MI, USA) and IgE (Bethyl Laboratories Inc., Montgomery, TX, USA) were measured by ELISA, according to the manufacturers' instructions.

### 2.13. Statistical Analysis

The data were reported as means ± standard error of the mean (SEM) and compared by ANOVA and the Bonferroni multiple comparison method. A *P* value <0.05 was defined as statistically significant. All statistical analyses were performed using the SYSTAT 8.0 program (SYSTAT Inc., Evanston, IL, USA).

## 3. Results

### 3.1. High-Performance Liquid Chromatography (HPLC) Analysis

A chromatogram of AKH extract was obtained using an HPLC-photodiode array (PDA) detector. Under optimized chromatography conditions, three compounds were eluted before 40 min in the sample analysis using mobile phases comprising solvent A (H_2_O) and solvent B (acetonitrile). 

The linearity of the peak area (*y*) versus concentration (*x*,  *μ*g/mL) curve for each component was used to calculate the contents of the main components in AKH. The correlation coefficients (*r* ^2^) of the calibration curves for three main constituents were >0.9999. Line equations and *r* ^2^ of values for the calibration curves are summarized in Table 1. The retention times of the compounds were 16.78 min (alpinetin), 25.37 min (pinocembrin), and 29.28 min (cardamonin).  Figure 1(b) shows the HPLC chromatogram of standard solution and AKH extract. The contents of three constituents, alpinetin, pinocembrin, and cardamonin were 5.28–6.38 mg/g, 7.57–9.16 mg/g, and 1.38–1.59 mg/g, respectively. The analytical results for each component identified are summarized in Table 2.

### 3.2. AKH Extract Inhibits NO and PGE_2_ Production in Lipopolysaccharide (LPS)-Stimulated RAW 264.7 Cells

**Figure 2:**
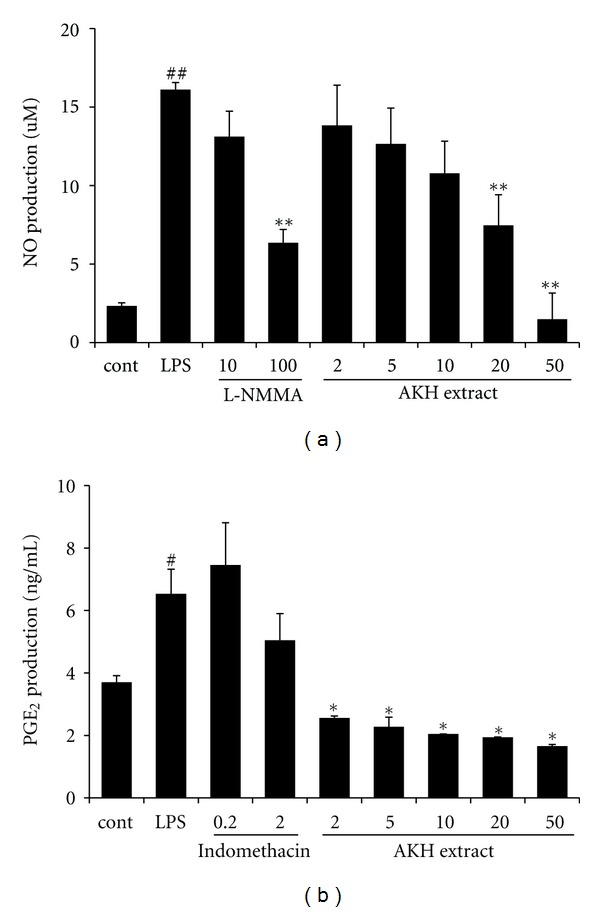
Effect of AKH extract on LPS-stimulated NO and PGE_2_ production in RAW 264.7 cells. The production of NO (a) and PGE_2_ (b) was measured in culture medium of cells treated with AKH extract (2–50 *μ*g/mL) and then costimulated with LPS (1 *μ*g/mL) for 18 h. L-NMMA (10 and 100 *μ*M) and indomethacin (10 and 100 *μ*g/mL) were used as positive control drugs. Each bar represents the mean of three independent experiments. ^##^ *P* < 0.01 versus vehicle control group; ^∗,∗∗^ *P* < 0.05 and *P* < 0.01 versus LPS-treated cells, respectively.

We first measured the cytotoxic effect of AKH extract on RAW 264.7 cells, exposed to various concentrations ranging from 2 to 200 *μ*g/mL of the AKH extract for 24 h. Cell viability was then measured using the CCK-8 assay. Nontoxic concentrations of the test materials were used for the subsequent experiments (data not shown). To determine the effects of AKH extract on NO and PGE_2_ production in LPS stimulation, RAW 264.7 cells were treated with different concentrations of AKH extract (2, 5, 10, 20, and 50 *μ*g/mL) and then stimulated with LPS (1 *μ*g/mL) for 18 h. AKH extract suppressed LPS-stimulated NO production in a dose-dependent manner (Figure 2(a)). LPS greatly stimulated NO production by RAW 264.7 cells (16.10 ± 1.623 nM). By contrast, AKH extract significantly decreased NO production to 7.47 ± 1.945 nM (*P *< 0.01) at a dose of 20 *μ*g/mL and to 1.498 ± 1.654 nM (*P *< 0.01) at a dose 50 *μ*g/mL. PGE_2_ production was higher in LPS-stimulated RAW 264.7 cells than in untreated control cells, whereas AKH extract inhibited PGE_2_ production (Figure 2(b)).

### 3.3. AKH Extract Inhibits TARC and Histamine Production in HaCaT Cells and HMC-1 Cells, Respectively

**Figure 3:**
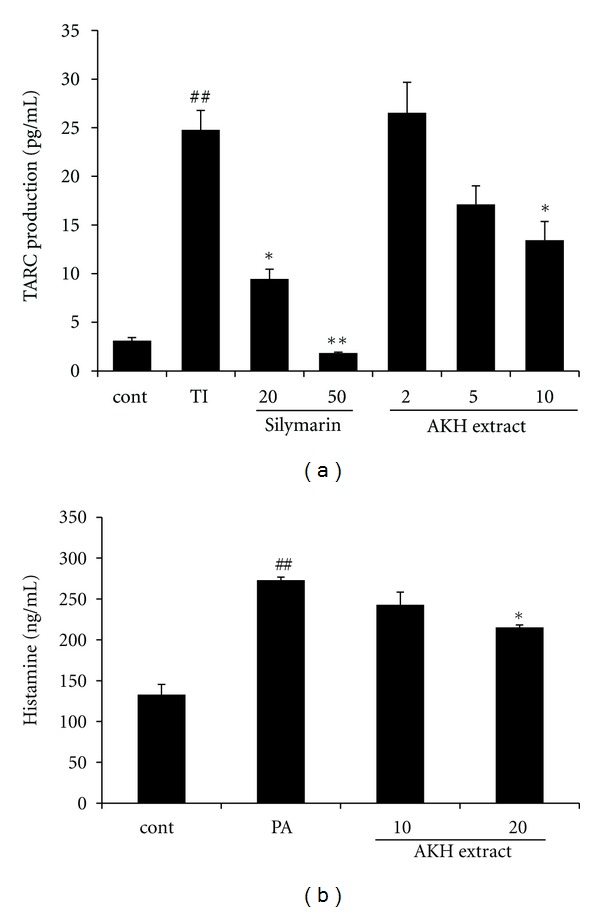
Effects of AKH extract on TARC and histamine production in HaCaT cells and HMC-1 cells, respectively. The production of TARC (a) was measured in culture medium of cells treated with AKH extract (2, 5, or 10 *μ*g/mL) and then costimulated with TNF-*α* and IFN-*γ* (each 10 ng/mL, TI) for 24 h. Silymarin (20 and 50 *μ*g/mL) was used as a positive control drug. The production of histamine (b) was measured in culture medium of cells treated with AKH extract (10 and 20 *μ*g/mL) and then costimulated with PMA (50 nM) and A23187 (1 *μ*M) for 24 h. Each bar represents the mean of three independent experiments. ^##^ *P* < 0.01 versus vehicle control group; ^∗,∗∗^ *P* < 0.05 and *P* < 0.01 versus TI-treated cells, respectively.

The effects of AKH extract on TARC production were assessed in TNF-*α* and IFN-*γ*-stimulated HaCaT cells. The cells were treated with different concentrations of AKH extract (2, 5, and 10 *μ*g/mL) and then stimulation with TNF-*α* and IFN-*γ* (10 ng/mL, TI) for 24 h. AKH extract suppressed TI-stimulated TARC production in a dose-dependent manner (Figure 3(a)). TI-treated cells produced significantly more TARC (24.78 ± 2.005 ng/mL, *P *< 0.01) than the control cells. The levels were reduced to 13.43 ± 1.93 ng/mL (*P *< 0.01) by AKH extract at 10 *μ*g/mL. In histamine production, PMA and A23187 (each 50 nM and 1 *μ*M, PA) stimulated cells (272.97 ± 3.71 ng/mL, *P* < 0.01) more increased than the controls. In contrast, AKH treated cells showed significant reduction (215.15 ± 2.94 ng/mL in 20 *μ*g/mL, *P* < 0.05) in histamine production compared with the PA stimulated cells (Figure 3(b)).

### 3.4. AKH Extract Suppresses the Severity of Dermatitis in Skin Lesions in Biostir-Ad Ointment-Induced AD in NC/Nga Mice

**Figure 4:**
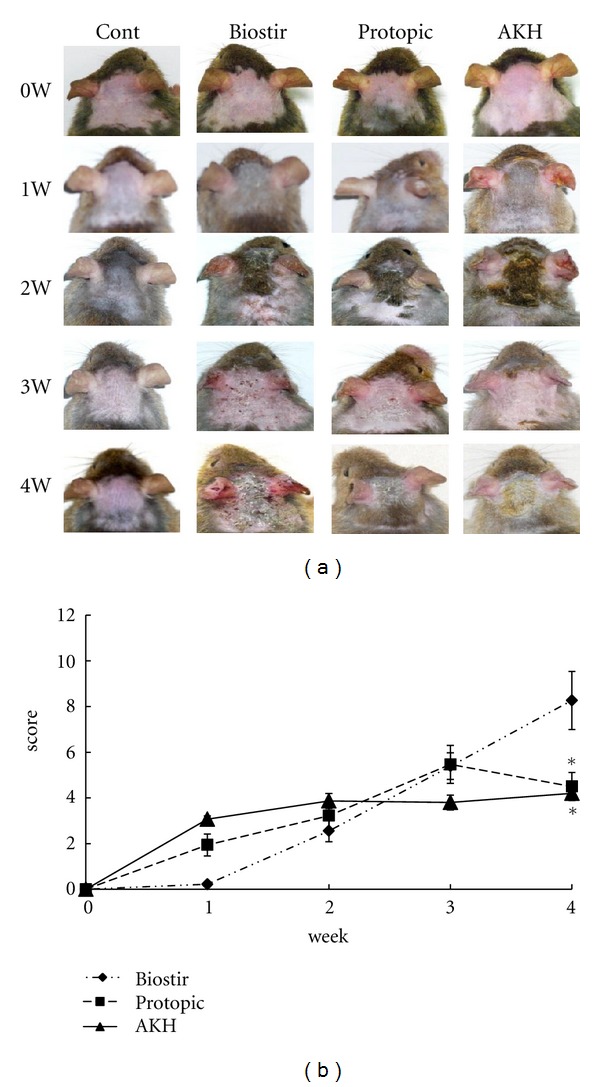
Effects of AKH extract on the development of AD induced by house dust mite-induced AD in NC/Nga mice. NC/Nga mice were used as a model of house dust mite induced AD. The mice were divided into four groups: untreated (Cont), Biostir-AD plus vehicle treatment (Biostir), Biostir-AD plus Protopic treatment (Protopic), and Biostir-AD plus AKH extract treatment (AKH). Clinical features (a) and dermatitis scores (b) were assessed using the criteria described in Section 2. The features were monitored once a week for 4 weeks. Values are expressed as the means ± SEM (*n* = 5). *P < 0.05 versus Biostir-AD treated mice.

The severity of dermatitis was evaluated once a week. Major clinical signs and symptoms developed shortly after the mite antigen was applied to the backs of the mice, and the AD-like skin lesions worsened progressively for 4 weeks after the initial treatment. Repeated application of Biostir-AD ointment in control mice induced skin dryness first followed by mild erythema, hemorrhage, and edema (Figure 4(a)). Finally, the skin became thick, and severe erythema, hemorrhage, edema, scarring, erosion, and excoriation were observed. The application of AKH extract inhibited the appearance of these skin symptoms. The sum of the individual scores for each symptom was taken as the dermatitis score (Figure 4(b)). In the Protopic-treated group, symptoms increased for 3 weeks but decreased thereafter. In the AKH extract-treated group, the dermatitis score was much lower at 3 weeks than that of the Protopic-treated group. These results indicate that AKH extract suppressed the spontaneously induced dermatitis in NC/Nga mice.

### 3.5. AKH Extract Attenuates AD-Like Lesions Induced by Biostir-AD as Shown by Histopathological Examination

**Figure 5:**
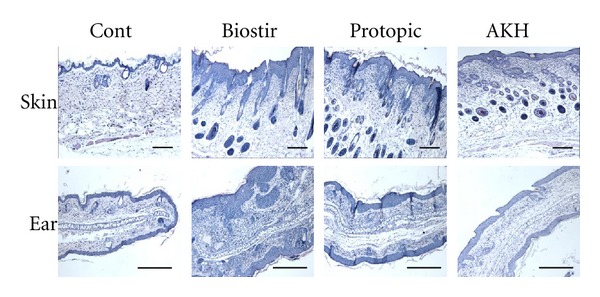
Effect of AKH extract on histological features of house dust mite-induced AD in NC/Nga mice.The skin and ear tissues of untreated (Cont), Biostir-AD plus vehicle-treated (Biostir), Biostir-AD plus Protopic-treated (Protopic), and Biostir-AD plus AKH extract-treated (AKH) mice were excised, fixed with 10% formaldehyde, and embedded in paraffin, and thin sections were made. The histological features of the skin and ear lesions were stained with hematoxylin and eosin and the back skin sections were stained with toluidine blue. Scale bars: 100 *μ*m.

Specimens from the back skin and ears of NC/Nga mice were examined histopathologically. As shown in Figure 5, Biostir-AD-treatment group showed the increased thickness of stratum corneum induced by hyperkeratosis and dermal edema with inflammatory cell infiltration. These pathological alterations are observed in both back skin and ears from Biostir-AD-treated group. In contrast, AKH extract-treated mice showed the reductions in thickness of stratum corneum and inflammation cell infiltration into dermis compared with Biostir-AD-treated group.

### 3.6. AKH Extract Inhibits IgE and Histamine Level in Biostir-AD-Induced AD in NC/Nga Mice

**Figure 6:**
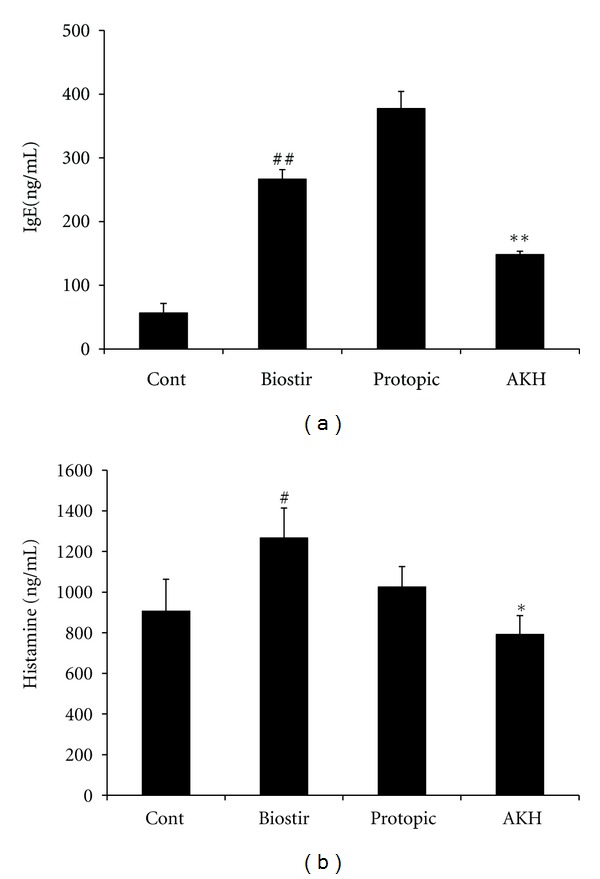
Effect of AKH extract on plasma level of IgE and histamine in house dust mite-induced AD in NC/Nga mice.The level of IgE and histamine were measured in plasma samples from untreated (Cont), Biostir-AD plus vehicle-treated (Biostir), Biostir-AD plus Protopic-treated (Protopic), and Biostir-AD plus AKH extract-treated (AKH) mice. The plasma concentrations of IgE (a) and histamine (b) were measured by ELISA. The values are expressed as the means ± SEM (*n* = 5). ^#,##^ *P* < 0.05 and *P* < 0.01 versus control group; ^∗,∗∗^ *P* < 0.05 and *P* < 0.01 versus Biostir-AD treated mice, respectively.

We measured the plasma levels of total IgE and histamine in NC/Nga mice. The IgE level was higher in Biostir-AD-treated group (266.53 ± 14.92 ng/mL) than in the control group (56.67 ± 14.91 ng/mL) (*P* < 0.01). By contrast, the IgE level was lower in the mice treated with the AKH extract (148.23 ± 5.24 ng/mL) than in the Biostir-AD-treated group (*P* < 0.01) (Figure 6(a)). The histamine level was lower in the AKH extract-treated group (791.43 ± 93.27 ng/mL) than in the Biostir-AD-treated group (1266.68 ± 147.24 ng/mL) (*P* < 0.05) (Figure 6(b)). These results demonstrate that treatment with AKH extract prevented the development of dermatitis in NC/Nga mice.

## 4. Discussion

Many previous studies on herbal medicines have been conducted to find the potential natural AD products through *in vitro* and *in vivo* systems. AKH is one of the important components of herbal medicine and has been used to treat various diseases such as antiemetic and a stomachic since ancient times [14].

Atopic dermatitis is characterized by a chronic inflammation skin disease. Inflammation response is mediated by several proinflammatory factors such as NO, PGE_2_, and TARC. NO and PGE_2_ induced by iNOS and COX2, respectively, which promoted inflammatory responses. In addition, TARC is known as chemotactic factor and accelerated the inflammatory cells infiltration into pathological lesion such including inflammatory lesion. In this study, we investigated the effects of AKH extract on atopic dermatitis by which AKH extract inhibits LPS-induced proinflammatory mediator production in RAW 264.7 macrophage cells, cytokine-induced Th2 chemokine expression in HaCaT human keratinocyte cells and histamine level in HMC-1 human mast cells. Moreover, we evaluated the effects of AKH extract on skin inflammation in NC/Nga mice. In NC/Nga mice, AKH extract significantly suppressed the development of house dust mite induced AD-like skin lesions. 

Activated macrophages produce a large amount of NO and PGE_2_ via an iNOS-driven COX-2-mediated pathway, as well as pro-inflammatory cytokines. At physiological concentrations, NO plays an important role as an immune regulator and vasodilator in a variety of tissues. However, at high levels, NO produced by iNOS is considered a cytotoxic molecule that is involved in inflammation and endotoxemia [16]. Like NO, PGE_2_ is a pleiotropic mediator produced by the COX-2 pathway at sites of inflammation, where it contributes to pain, swelling, and stiffness [17]. We showed that AKH extract reduced NO and PGE_2_ production in a dose-dependent manner in LPS-stimulated RAW 264.7 cells.

The infiltration of inflammatory cells into sites of inflammation is dependent on the local production of various members of the chemokine family with leukocyte chemoattractant activity [18]. TARC functions as a selective chemoattractant and assists in the recruitment and migration of Th2 cells, which express CC chemokine receptor 4 [19]. TARC is overexpressed by keratinocytes, especially, epidermis in atopic dermatitis lesions in both murine model and atopic dermatitis patients [20]. Therefore, TARC may be an important mediator that exacerbates AD. In this study, we examined the effect of AKH extract on TARC production in TI-stimulated HaCaT cells. We used silymarin as a positive control to suppress inflammation. The pharmacological efficacy of AKH extract in inhibiting AD might be associated with its dose-dependent inhibitory actions on TARC production in TI-stimulated HaCaT cells.

AD is the most common skin disease worldwide [21], and the incidence of AD continues to increase in industrialized countries [7]. To evaluate better the biological parameters of AD, NC/Nga mice are used as an experimental animal model. NC/Nga mice have been used widely in previous studies of AD because this animal model manifests many clinical traits characteristic of AD, including increased serum IgE and histamine levels [22]. IgE production is one of the most important therapeutic targets in AD because high serum IgE levels mediate the critical clinical characteristics of atopic diseases. Specifically, IgE binds mast cells causing the release of inflammatory mediators, whose levels correlate with AD severity [23]. Histamine, serotonin, and substance P are regarded as mediators of itching in humans [24]. We found that repeated application of Biostir-AD in NC/Nga mice induced hemorrhage, edema, and erosion in skin lesions and a significant increase in plasma levels of IgE and histamine. We evaluated the effects of AKH extract on skin inflammation in these AD mice. In NC/Nga mice, topical application of Biostir-AD-induced AD-like skin lesions, including hemorrhage, excoriation, and dryness, and AKH extract significantly suppressed the development of these symptoms. Histological analysis demonstrated less infiltration of leukocytes into the back and ear lesions after AKH extract treatment. These results indicate that AKH extract has therapeutic effects in the AD mouse model.

We also used HPLC-PDA to analyze the content of the three major components in AKH extract, alpinetin, cardamonin, and pinocembrin. Our result showed good separation of the AKH extract. Alpinetin has antibacterial, anti-inflammatory, and other important therapeutic activities at significant potency [25]. Cardamonin has numerous biological roles, including an antitumour property, insecticidal effect, antimutagenic activity, and ability to inhibit the platelet aggregation induced by arachidonic acid, collagen, adenosine diphosphate, or ristocetin [26–29].

In conclusion, inhibition of the production of NO and PGE_2_ is responsible for the anti-inflammatory effects of AKH extract. These beneficial effects may be associated with the inhibitory effect of AKH extract on TARC production by keratinocytes in dermatitis lesions. AKH extract may also have moisturizing of dry skin and appears to mitigate the symptoms of AD. The topical application of AKH extract prevented the development of house dust mite-induced AD in NC/Nga mice. These findings provide evidence that AKH extract has potential as a therapeutic agent in the treatment of AD.

## Figures and Tables

**Table 1:** Calibration curves for the marker components (*n* = 3).

Component	Linear range (*μ*g/mL)	Regression equation^a^	*r* ^ 2^
Alpinetin	1.56–200.00	*Y* = 36,943.64*x* – 21,495.52	0.9999
Pinocembrin	1.56–200.00	*Y* = 32,328.64*x* – 21,267.92	1.0000
Cardamonin	0.78–100.00	*Y* = 102,355.01*x* – 27,440.91	0.9999

^a^ *Y* represents peak area (mAU); *x* represents concentration (*μ*g/mL).

**Table 2:** Contents of the three marker compounds in *A. katsumadai* (*n* = 3).

	Compound								
Batch (#)	Alpinetin			Pinocembrin			Cardamonin		
	Mean (mg/g)	SD	RSD (%)	Mean (mg/g)	SD	RSD (%)	Mean (mg/g)	SD	RSD (%)
1	6.38	0.002	0.031	9.16	0.154	1.678	1.59	0.009	0.561
2	5.99	0.011	0.176	8.57	0.128	1.491	1.57	0.020	1.254
3	5.28	0.005	0.100	7.57	0.195	2.577	1.38	0.023	1.647
